# “In Our Own Little World”: Invisibility of the Social and Ethical Dimension of Engineering Among Undergraduate Students

**DOI:** 10.1007/s11948-021-00355-0

**Published:** 2021-12-09

**Authors:** Jae Hoon Lim, Brittany D. Hunt, Nickcoy Findlater, Peter T. Tkacik, Jerry L. Dahlberg

**Affiliations:** 1 Cato College of Education, The University of North Carolina at Charlotte, 9201 University City Blvd, , Charlotte, NC USA; 2 Lee College of Engineering, The University of North Carolina at Charlotte, 9201 University City Blvd, , Charlotte, NC USA

**Keywords:** Engineering ethics, Undergraduate students, Qualitative research

## Abstract

This paper explores how undergraduate students understood the social relevance of their engineering course content knowledge and drew (or failed to draw) broader social and ethical implications from that knowledge. Based on a three-year qualitative study in a junior-level engineering class, we found that students had difficulty in acknowledging the social and ethical aspects of engineering as relevant topics in their coursework. Many students considered the immediate technical usability or improved efficiency of technical innovations as the noteworthy social and ethical implications of engineering. Findings suggest that highly-structured engineering programs leave little room for undergraduate students to explore the ethical dimension of engineering content knowledge and interact with other students/programs on campus to expand their “technically-minded” perspective. We discussed the issues of the “culture of disengagement” (Cech, Sci Technol Human Values 39(1):42–72, 2014) fueled by disciplinary elitism, spatial distance, and insulated curriculum prevalent in the current structure of engineering programs. We called for more conscious effort by engineering educators to offer meaningful interdisciplinary engagement opportunities and in-class conversations on ethics that support engineering students' holistic intellectual growth and well-rounded professional ethics.

## Introduction

Engineering ethics has been an integral part of the engineering profession worldwide for a long period of time (Hess & Fore, 2018; Institution of Engineers, Australia, 1997; Zandvoor et al., 2000). In 1947, American Engineers' Council for Professional Development (ECPD) adopted an ethics code declaring engineers' duty to serve for public welfare and to ensure the safety of life and health of the public (Mitcham, 2009). With the ECPD's repeated ethics code revisions over the next several decades, the ethical dimension of engineering work, including engineers' social and ethical awareness, has become an essential part of the profession’s discourse. At the turn of the century, the Accreditation Board of Engineering and Technology (ABET) listed “an understanding of professional and ethical responsibility” as one of its major outcomes (criterion 3f, ABET EC 2000), acknowledging the importance of systemic inclusion of ethics in engineering curriculum (Lattuca et al., 2006). Encouraged by the ABET's new criterion coupled with growing interest by the public and evolving discussions within professional societies, engineering ethics and engineers' social and ethical awareness have emerged as an important topic among scholars and educational researchers (Herkert, 2005; Hersch, 2017).

Despite the long, historical emphasis on engineering ethics, scholars still acknowledge that engineers consider themselves as effective problem solvers less inclined to ponder upon the wider significance of their work and its potential consequences (Herkert, 2001). Discussions on engineering ethics remain on the level of “engineers’ ethics,” personal ethics left to the individual’s discretion and actions (Basart & Serra, 2013) rather than addressing the profession’s collective responsibilities to society. There is no shortage of literature reporting multiple challenges in Teaching Engineering Ethics, confirming the hardship of raising the next generation of engineers equipped with a well-rounded professional ethics (Li & Fu, 2012; Martin et al., 2021). Scholars recognize that facilitating students’ critical awareness of and commitment to the profession’s shared responsibility to the welfare of larger society is one of the hardest tasks to accomplish through the programmatic and instructional means currently available in higher education (Martin et al., 2021). This calls for a fundamental perspective change in engineering education—including how to conceptualize, address, and teach engineering ethics—to discuss to ensure that the next generation of professional engineers is equipped with a robust, more holistic concept of engineering ethics.

Current literature on engineering ethics presents two major strands. One strand is devoted to the philosophical debates and conceptual refinement (e.g., Basart & Serra, 2013; Herkert, 2005; Mitcham, 2009); the other is focused on the pragmatic aspects of curriculum innovations and teaching strategies (e.g., Alpay, 2013; Chung, 2015; Li & Fu, 2012). Empirical studies on students’ understanding of engineering ethics are mostly based on survey research (e.g., Cech, 2014; Garibay, 2015; Stappenbelt, 2013) that may limit the depth of exploration and fail to convey the authentic viewpoints of the students. A limited number of qualitative studies were conducted in a stand-alone engineering ethics class (e.g., Alpay, 2013; Hess et al., 2017) or an experimental context (Lee et al., 2017) where students were clearly aware of the objectives of the course/experiment, the instructor’s expectations, and subsequent evaluation directly tied to engineering ethics. Few empirical studies illuminate engineering students’ understanding of engineering ethics based on the students’ first-hand accounts, and what they consider as problems (or non-problems) and possible solutions in a typical engineering class where they can unravel their authentic perspectives and attitudes toward the topic without feeling the pressure to show the engineering ethic-related competencies expected by the instructor. This void of student perspective on engineering ethics situated in most engineering classes creates a significant gap in our understanding of this critical issue and makes it hard for engineering educators to properly grasp the scope of students’ ethical apathy reported in previous survey studies (Cech, 2014; Garibay, 2015; Stappenbelt, 2013). This void also makes it difficult to assess why it is hard to facilitate students’ ethical awareness through everyday teaching of engineering content knowledge.

Scholars contested that the current challenges in Teaching Engineering Ethics is deeply grounded in the culture of engineering which over-stresses technical knowledge while marginalizing the ethical and societal dimension of engineering (Jamison et al., 2014; Schiff et al., 2021). Students’ perceptions are shaped by the binary cultural frame. They classify the engineering courses covering technical content as “real engineering” (Polmear et al., 2018) and discount the importance—even the necessity—of discussing engineering ethics (Lönngren, 2021). While this binary conceptualization may be most prevalent in the typical, technical content focused engineering courses, those courses, we contest, are also a critical space where students’ authentic perspectives on engineering ethics can be unraveled and engineering educators can —if they choose to do so—challenge the students’ binary conceptualization by showing the inevitable connection between the course’s technical/scientific curriculum and engineering ethics.

Over the past two decades, engineering education researchers have offered significant insights about engineering students’ ethical reasoning (e.g., Hess et al., 2017) and promising instructional strategies (Alpay, 2013; Lee et al., 2017) in a stand-alone engineering ethics course(s) with service-learning components (Pritchard, 2000). However, it should be noted that most engineering courses cover technical and scientific knowledge; students develop their professional engineering competencies and identities in the cultural context of those courses. Therefore, gaining an accurate and in-depth understanding of student perspective in the technical curriculum-focused engineering classes is critical to the development of an innovative strategy that helps overcome the unproductive, yet persistent cultural and instructional divide between what students consider “real” engineering and “fuzzy” ethics (McGinn, 2003) and facilitate students’ growth in professional engineering ethics throughout the entire spectrum of engineering coursework.

To address this void in the existing literature, this study explores how undergraduate engineering students understand the ethical dimension of their engineering course content knowledge that focuses on various technologies for military purposes. The study examines how these students identify, evaluate, or fail to evaluate the larger social and ethical implications from their technical engineering knowledge. Further analysis also scrutinizes how these students make sense of peers’—and their own—lack of understanding and exposure to the ethical dimension of engineering as they reflect on its causes. This study is guided by three interrelated research questions:

- How do undergraduate engineering students understand the larger social and ethical implications of their engineering course content?
- How are the two dimensions of engineering ethics, micro and macroethics, reflected in their understanding of engineering ethics?
- How do they make sense of peers’—and their own—lack of understanding and exposure to the ethical dimension of engineering in their critical reflections?

## Literature Review

Our study is grounded in three strands of scholarly literature, which address the three key components of the study, teaching engineering ethics, student perceptions, and engineering cultural context. The first sub-section explains the history and current practices of teaching engineering ethics and pedagogical challenges. The second sub-section offers a critical synthesis of empirical research on students’ perceptions and learning experiences in various instructional contexts. We also introduce a key theoretical concept, “micro and macroethics,” which illustrates tension and void in current engineering ethics education and, therefore, invites a new, critical, and culturally informed perspective. The last sub-section presents a summary of recent studies focused on the cultural context of teaching engineering ethics and proposed solutions gleaned from those studies. These three strands of literature offer a solid scholarly ground for our research that investigates students’ authentic perceptions of ethics in the context of a technical engineering course from a critical cultural perspective.

### Teaching Engineering Ethics

Near the turn of the twenty-first century, the institutions of higher education around the globe started to incorporate ethics into their engineering curriculum prompted by societal demand, accreditation agencies’ new evaluation criteria, and state licensing policies (Chung & Alfred, 2009). In the United States, the ABET Engineering Criteria 2000, known as EC2000, was an important step forward as it required engineering programs to demonstrate that their graduates have an understanding of professional and ethical responsibility (Criterion 3.f) (Herkert, 2001; Lattuca et al., 2006). Likewise, professional or state licensing agencies implemented engineering ethics as an essential part of the state licensing exam (Chung & Alfred, 2009). To meet new accreditation criteria and licensing requirements, many educational institutions developed new instructional models on engineering ethics, assisted by researchers, utilizing various pedagogical and assessment methods to evaluate engineering students’ ethical awareness and efficacy in dealing with ethical decision making (Bairaktarova & Woodcock, 2016).

However, empirical studies provide a bleak picture of engineering ethics education at the program/curriculum level and in student outcomes. Despite the external push for engineering ethics, courses on this topic are rarely considered as a core requirement in the engineering curriculum, making it hard for students to take ethics seriously (Barry & Ohland, 2012; Colby & Sullivan, 2008). The proportion of the curriculum devoted to or addressing engineering ethics is minimal, reflecting its marginal status in a technically dominant curriculum (Fabregat, 2013; Miñano et al., 2017). Colby and Sullivan’s (2008) extensive review of engineering ethics curricula found that the ethics portion of the course requirements is rarely graded, which may lead students to discount its importance and consider it non-essential or irrelevant (Li & Fu, 2012).

Considering the peripheral position of engineering ethics curriculum, it is counter-intuitive to expect that students naturally gain a well-rounded perspective on engineering ethics and commit themselves to upholding a high moral stance in the profession. Not surprisingly, earlier research indicates an inconsistent growth pattern in engineering students’ ethical reasoning throughout the program (Shuman et al., 2004). Furthermore, students struggle most with an ethical dilemma that requires them to consider differing perspectives and determine an optimally balanced position. Research based on the Survey of Engineering Ethical Development (SEED) project indicates that engineering students made little progress in their understanding of ethics as measured by Fundamental Engineering (FE) type questions (Harding et al., 2013). Even though the effectiveness of FE type questions as the measure of engineering ethics is open to further discussion, the identified weakness—engineering students’ lack of ethical awareness—has been confirmed in multiple empirical research studies by scholars around the world (Bielefeldt & Canney, 2016; Cech, 2014; Garibay, 2015; Stappenbelt, 2013). For example, in her multi-institutional survey research, Cech (2014) confirmed that engineering students’ interest in public welfare waned over the four years of their undergraduate program. Bielefeldt and Canney (2016) reported a similar, disappointing pattern noting that most engineering students’ social responsibility attitudes remained stagnant or declined throughout their program years. Garibay (2015) also confirmed that engineering students’ sense of social agency (importance of working for social change) significantly declined through higher education, which suggests the impact of academic majors and peer influence in the undergraduate socialization process. Furthermore, engineering students are more likely to retain a narrow and rigid understanding of ethics (Culver et al., 2013; Lee et al., 2020), which contradicts the growing consensus in the profession focusing on engineers’ broader and participatory social responsibility.

### Student Perceptions of Engineering Ethics

While most studies on engineering students’ professional ethics development and innovations in engineering ethics education are quantitative (Bielefeldt & Canney, 2016; Cech, 2014; Garibay, 2015; Stappenbelt, 2013), a limited number of qualitative studies exist offering a further, more in-depth, insight into student experiences and perceptions of engineering ethics in varied program and course contexts. Loui’s qualitative study (2005) indicates that engineering students learn about professionalism mainly from their engineer relatives and co-workers but rarely from their technical engineering courses. Alpay’s study (2013) confirms that first-year engineering students are already aware of the marginal status of ethics in their programs based on the lack of attention to the ethical aspects of their technical course content knowledge. A first-year undergraduate student acknowledged, “[in] a Computing degree we don’t always appreciate the seriousness of what we are doing and the ethical implications of it” (p. 1463). The participant noted her engineering ethics course was at odds with other engineering courses that pay little attention to ethical issues. Hashemian and Loui’s study (2010) shows that engineering students who did not take an elective engineering ethics class exhibited minimal sense of responsibility as they responded to an engineering ethics-related case scenario considering the posed problem as “not my business.’’.

It is noteworthy that most qualitative studies on engineering ethics have explored the teaching and learning process, student experiences and perceptions, and most importantly, the outcomes of the instructional innovation implemented in a stand-alone engineering ethics course or other intentionally-designed instructional contexts. Therefore, those studies accumulated a significant knowledgebase on teaching engineering ethics, including the characteristics of ethics teaching/learning process and the effectiveness of a specific approach on student learning outcomes. For example, Loui (2005) identifies that engineering students expand their perspectives on engineering ethics and increase self-confidence in moral reasoning in a class where they examine the cases of actual incidents and engaged in open dialogues with peers. She reports that some students—though not the majority—show a deeper understanding engineering ethics and professional responsibilities that goes beyond individual liability for blame but encompasses the role of stewardship for society. Hashemian and Loui’s (2010) study confirms the positive impact of a stand-alone engineering ethics’ course on students’ overall sense of responsibility and agency: Students who took an engineering ethics course are more likely to express a stronger sense of responsibility and engage in corrective action than those who did not take the course.

Qualitative research has also helped identify key internal and external factors contributing to the positive learning outcomes in engineering ethics courses. Alpay (2013) explains that students’ sense of creativity and agency are pivotal to effective teaching of engineering ethics. He explains that when students are given the role of creating strategies to teach engineering ethics to peers, they become more proactive in formulating their actions in a situation they expect to have soon (e.g., choosing summer work placements, evaluating the ethical dimension of their future employers). Hess et al. (2017) also highlight the importance of real-life connection in students’ sense-making and reflections. Students who could connect the ethical scenario to their own everyday work context or professional careers were able to grasp the complexity of the situation with multiple stakeholders and draw a deeper reflection. Furthermore, students’ diverse backgrounds yield contrasting perspectives, which helps generate cognitive dissonance and demand the need for open-mindedness.

While recognizing the significant contribution made by prior qualitative research studies in stand-alone engineering ethics courses or a similar experimental context, it is important to note that the prevailing goals of those courses tend to target individuals’ ethical awareness, perspective-taking, ethical decision-making skills, and commitment as major learning outcomes (Hess & Fore, 2018). As a result, the type of professional engineering ethics pursued in most courses remains within the scope of “microethics” (Herkert, 2001) leaving other critical dimensions of engineering ethics behind. Martin et al. (2021) note that the invisibility of macro topics in engineering ethics education including the broader mission and implications of the profession and public good.

The concepts of “microethics” and “macroethics” were initially articulated by ethicist John Ladd (1980) to clarify two different yet interrelated sub-domains of engineering ethics. In each domain, Ladd explains that the focus of microethics lies in the relationships between individual engineers and their clients, colleagues, and employers; macroethics addresses the profession’s external relationship with and shared responsibility in greater society. Herkert (2001) identified three frames of interests—individual, professional, and social—in the discussion of engineering ethics, and highlighted the importance of incorporating micro- and macro-level ethics education in engineering curriculums. By merging Ladd’s (1980) and Vanderburg’s (1995) terminologies, he conceptualized “microethics” as the term focusing on concerns related to the individual and the inner-workings of the engineering profession, whereas “macroethics” is defined as the collective social responsibility of the engineering profession and societal concerns about technology. As scholars began to address the issues of incorporating micro and macro-level ethics education in engineering curricula, Herkert identifies a need to bridge these approaches to improve the quality of engineering ethics education. Herkert argues that De George’s concept of “ethics of engineering” (1981) bridges Ladd’s and Vanderburg’s terminologies of micro- and macro-level ethics by highlighting that professional engineering societies have the potential to go beyond their role of only establishing and enforcing codes of ethics, but to also engage in proactive consideration of the broader impacts of technology in society. Herkert’s notion of engineering professional ethics that emphasizes the role of engineering professional societies to encompass both micro and macroethics is noteworthy considering the persistent ideological dualism (e.g., technical and social competencies) prevailing in the field (Cech, 2014). Scholars recognize that engineering macroethics—focusing on the profession’s collective responsibility to the welfare of larger society—is indeed the weakest area in the current discussions and practices of engineering ethics education.

### Cultural Context of Teaching Engineering Ethics

The growing body of empirical research over the past two decades suggests that systemic and cultural level barriers may explain the unsuccessful implementation of ethics in engineering education programs (Martin et al., 2021). Understanding the larger cultural and institutional context of technically dominant engineering education programs seems essential to understanding the current challenges in Teaching Engineering Ethics to undergraduate students. Cech (2014) contested that the “culture of disengagement” prevails in engineering programs hampering undergraduate students’ interest in and commitment to public welfare. She explains three ideological backdrops of the culture: the concept of depoliticization, which portrays non-technical matters irrelevant to engineering, and technical and social dualism, which discounts non-technical and social competencies, and meritocracy which uphold the current social structure as natural, legitimate, and fair. In their extensive review of research on engineering ethics education, Martin et al. (2021) argue that the culture of engineering education is characterized by “the valorisation of the technical and the marginalisation of the societal dimension of engineering” (2021, p. 60). Researchers emphasize a persistent cultural bias that evaluates non-technical knowledge and competencies as holding less value and importance in engineering fields (Niles et al., 2020, p. 498). Engineering ethics is not viewed as an integral part of engineering but rather considered as “something other,” separated from the dominant technical curriculum in engineering education (Lönngren, 2021; Polmear et al., 2018). Students conceptualize ethics a non-essential, even irrelevant component in engineering education (Lönngren, 2021). Tormey et al. (2015), highlight the enormous difficulty—though not impossibility—of facilitating students’ ethical reasoning in the current adversarial cultural context by referring it as “swimming against the hidden cultural tide of the programme as a whole” (p.2).

Scholars also suggest that the lack of cultural diversity plaguing most—though not all—engineering disciplines could be a significant hurdle in supporting students’ ethical awareness and commitment. Previous studies indicate that individuals from diverse backgrounds and life experiences bring in different perspectives creating a fertile ground for deeper reflections and perspective changes (Hess et al., 2017). Racial and ethnic minority students are more aware of ethics and moral principles based on their first-hand experiences with social inequity (Thoman et al., 2015), which may prompt them to develop a strong ethical stance to challenge the observed social injustice (Naphan-Kingery et al., 2019). Bielefeld et al. (2018) explain that women and underrepresented minority instructors are more likely to include social justice-related topics in their courses and/or cocurricular settings than their male counterparts. When engineering programs remain white-male-dominated in both faculty and student representation, diversity is often deemphasized, unsupported, or discouraged (Johnson, 2019). The dearth of diversity in engineering programs squarely contradicts its professed commitment to public welfare since underrepresented students of color (USC) “have significantly higher aspirations to work for social change than their non-USC counterparts (Garibay, 2015, p. 626).”

The National Research Council (2010) recommends that engineering curricula should incorporate social and humanitarian issues to effectively facilitate students’ awareness and understanding of social justice issues. Larsen et al. (2017) identify the interdisciplinary nature of the engineering profession and recommend that students must be provided opportunities to work with peers outside of the engineering program to develop various non-technical, yet important professional competencies and skill sets. Research shows that teams with cognitively diverse problem-solving skills outperform teams considered to be the best problem-solvers, highlighting the importance of diversity over ability or skill (Atadero et al., 2018; Page, 2007). Various curricula innovations that facilitate interdisciplinary collaboration or strengthen community connection have shown to have a positive impact on students’ ethical reasoning skills (Lee et al., 2020) and commitment to civic activities (Bielefeldt & Canney, 2016; McClure & Lucius, 2010; Middlecamp et al., 2006). Because today’s engineering profession has become increasingly globalized, it seems inevitable for engineering education programs to pay more attention to how to better support the holistic development of future engineers, so they are equipped with balanced competencies and perform optimally and ethically in the field (Larsen et al., 2017). Engineers’ awareness of and commitment to the public welfare should be promoted as one of key professional developmental goals in engineering education (Cech, 2014).

While acknowledging the benefits of community-linking extracurricular activities and stand-alone engineering ethics courses, another critical space for engineering ethics education is undoubtedly the typical technical content-focused engineering courses (Hess & Fore, 2018), which occupy the large segment of the engineering curriculum that are considered as the core courses by students. Davis (2006) contests that incorporating engineering ethics issues into technical engineering courses across the entire engineering curriculum, would be desirable in helping students understand the omnipresence of ethical issues in the engineering profession. Psychological studies (Colby & Damon, 1993) suggest that individuals’ conviction on an ethical judgement aligns with their identities. Hashemian and Loui (2010) rightly point out that aligning engineering ethics to students’ emerging professional identity is key in facilitating their moral courage and commitment “to choose the right action even in everyday situations” (p. 211). Understanding students’ authentic perspective on engineering ethics in the courses in which they are exposed to technically dominant curricula, and most importantly shape their professional identity as engineers, is an important steppingstone toward challenging and dismantling the persistent binary of “real engineering” and ““something other,” embedded in engineering programs in the United States and around the globe.

## Methods

Our research is a three-year qualitative study conducted in a junior-level engineering class at a large public university in the Southeast. The university is a co-educational urban research institution with approximately 29,700 students enrolled in 2018. The overall undergraduate student demographics are moderately diverse consisting of 57% Caucasian, 16% African American, 10% Hispanic, 7% Asian/Pacific Islander, 5% multiracial, 1% American Indian, 2% other/unknown, and 3% international students. The College of Engineering has over 3,500 enrolled students including 69 students receiving GI benefits in 2018.

ENGR 3999 (a pseudonym), an elective course available to all engineering majors, was a unique—and possibly ideal—class for our investigation on engineering ethics. This was a typical engineering course covering technical curriculum, which was designed to give both veteran and non-veteran students the opportunity to gain knowledge on military technology applications and science/technology career opportunities. ENGR 3999 focused on theories, research, and applications of various technologies frequently used for military purposes or in military contexts while allowing students to work in related experimental labs that involve configuring instrumentation as well as data acquisition and analysis from experiments. The course instructor and TAs did not explicitly engage the students in any discussion of ethical issues during the semester. However, they carefully designed the course contents around non-lethal technologies like parachutes, rocket and jet engines, high-speed vehicles, radio communication, and research tools. Because this was a junior-level class, students had completed another introductory engineering class that covered engineering ethics and engineers’ social responsibilities.

Data for this study were obtained from 71 students who were enrolled in the ENGR 3999 course in the fall semester of 2017, 2018, or 2019. The course enrollment was limited to 20–30 students. Only one section was offered each fall semester, which resulted in three-year-long data collection to accumulate enough data for meaningful analysis. The 2017 class included 31 students, the 2018 class included 20 students, and the 2019 class had 21 students. The engineering students enrolled during the three years were all upperclassmen (i.e., juniors and seniors). Out of the 71 study participants, 60 were male (85%). Of these 60 male students, 49 were Caucasian. The other 11 students identified as members of racial/ethnic minorities and included five African Americans, five Asians, and one Hispanic/Latinx. Of 11 female students, there were seven Caucasians, three Asians, and one African American. Nine male students were student veterans that accounted for 12.7% of the class. The course instructor for all three years was a Caucasian male. Two teaching assistants were Caucasian males (2017 to 2018), one of whom was a veteran, and one teaching assistant in 2019 was African American male who was a student veteran.

This study is based on two sets of qualitative data, 16 focus group interviews in which 66 students (92%) participated, and student reflective journal writings that all 71 students submitted throughout the course. One of the students’ reflective journal prompts and the focus group interview protocol included questions probing their perspective about engineering ethics. To avoid normative responses from students, we decided not to use the term “engineering ethics” directly but used slightly different phrases to draw as honest as possible responses from students regarding the relevance of the course work to the profession’s broad ethical responsibilities to society. Students submitted four reflective writing assignments during the semester; each featured four to six open-ended questions. Students were free to write a response of any length to each open-ended question. The final reflective journal prompt had an open-ended question that probed their stance on engineering ethics. (“Have the knowledge and skills you have learned in this course impacted/changed your view regarding the roles of engineers and engineering knowledge in the larger society and for various groups of people? If so, how?”). The length and depth of student responses to the question varied, yet a typical response was one solid paragraph between 60 and 120 words. Students’ reflective journals were also compiled and provided to the research team for analysis.

All 16 focus group interviews led by a member of the research team were conducted at the end of the semester. Each focus group typically included 3–5 students with three exceptions (one 2-member focus group in 2017 and two 6-member groups in 2019). Interviews lasted between 20 to 35 min. The focus group interview protocol included several key questions about the students’ motivation to enroll in the class and their course experiences in addition to the engineering ethics question. (“How do you think the knowledge and skills obtained in this class can be related or applicable to some important contemporary social issues or some humanitarian causes?”) We intentionally chose focus group interviews—rather than individual interviews—considering the need to bring in the course context as the starting point for dialogue. In this study, we conceptualize students’ perceptions of and attitudes toward engineering ethics as a cultural phenomenon situated in the technical engineering course context. In addition to the sociocultural alignment between the course environment and interview context, a focus group context with classmates offered further benefits since the format encouraged students to freely express their opinions while recognizing peers’ consensus and support. We expected the final reflective writing assignment would generate each student’s *individual* perspective. Having two types of data, one from an individual perspective and the other revealing the shared cultural pattern among students would, we believe, complement each other, and offer a holistic portrayal of the topic under investigation. All 16 focus group interviews were transcribed verbatim by a member of the research team and immediately reviewed by the interviewer to ensure accuracy. All data collection activities in the course, including the focus group interviews and student journals, were approved by the university’s Institutional Review Board in advance of the study. Students enrolled in the course were informed by the instructor early in the semester that our evaluation team would routinely collect various data from the class including their reflective journal assignments. In addition, students were informed of their right not to participate in the end of semester focus group interviews without negative consequence.

The research team included five researchers, each possessing a different cultural, ethnic, and professional background. The research team was led by two faculty members, an Asian-American female who served as the qualitative evaluator for the course implementation and a Caucasian male engineering faculty member teaching the ENGR 3999 class. Three research assistants who were also part of the team included one Caucasian male Ph.D. student veteran in engineering, one Black male Ph.D. student with an engineering advising background, and one Native American female Ph.D. student in educational research.

Our data analysis followed the general guidelines of thematic analysis (Ezzy, 2002; Tracy, 2019). The lead investigator and two graduate students independently read the 16 interview transcripts and reflective journals and identified several significant themes recurring in the textual data. Our team met and discussed the points identified by each member, building consensus about the focus of the analysis, and establishing an initial set of open codes. NVivo, a qualitative analysis software, was used to ensure consistency and thoroughness in the coding process. We first imported data from the 16 focus group interviews and analyzed the data using the initial open code list. The code list was repeatedly revised based on newly identified inductive codes from the data and the team’s discussions during the subsequent meetings. Student reflective journal responses were added later and were coded using the same set of codes and categories developed from the focus group interviews. When focus group interview data and reflective journal entries were initially analyzed by the evaluation team, the lead investigator shared the preliminary findings with the course instructor and student veteran teaching assistant and asked for their input. In the later stage of analysis, the entire research team went back to literature and examined how our inductively-derived categories could be linked to and reinterpreted through theoretical concepts (micro/macroethics) available in the existing literature. We also situated our findings in the larger institutional and program contexts as the whole team finalized the major themes described in Findings. In summary, the data analysis was a three-year long collaborative and reiterative process by the highly diverse research team.

## Findings

The four major themes that emerged from an analysis of the interviews and reflective journals are: (1) Prevalence of microethics: Pragmatic usability and technical efficiency as social relevance of engineering, (2) Emerging macroethics discourse, (3) Apathy and disregard, and (4) Searching for why. The first three themes collectively answer our first and second research questions. The last theme addresses the third research question focusing on students’ critical reflections. Table 1 shows the frequencies and percentages of key codes and upper categories in the 16 focus group interviews and 71 student reflective journal entries. These themes are further explained below with supporting evidence and quotes from students.

**Table 1:** Percentages of categories in focus groups and reflective journal writing

Category	N (FG)*	% (FG)	Sub- categories	# of quotes (FG)*	# of students (RJW)**	% (RJW)
Microethics I: pragmatic usability	14	87.5%	Technical usability	45	20	28.2
			Career-wise usability	30	25	35.2
Microethics II: technical efficiency	11	68.6%		13	6	8.4
Emerging macroethics	13	81.2%	Humanitarian value	17	4	5.5
			Positive contribution to society	22	9	12.7
			Engineering ethics and perspective change	16	21	29.5
Apathy and disregard	11	68.7%	Apathy and disregard	16	29	40.8
			No perspective change	2	27	38.0
			Technically minded people	6	0	0
Searching for why: critical questioning	7	43.8%		18	2	2.8

*N(FG): Number of the focus groups showing the corresponding code/category at least once

**Frequency (FG): Number of all quotes coded in all 16 focus group transcripts

***N(RJW): Number of students whose reflective writing responses has the corresponding code

### Prevalence of Microethics: Pragmatic Usability and Technical Efficiency

Our analysis showed that students’ discussions of engineering ethics tended to focus on the issues falling under microethics (Herkert, 2001). These students interpreted “social relevance of engineering” as either pragmatic and immediate usability of technology, or improved efficiency of technological innovations (See Table 1). This suggests that the majority of students had difficulty envisioning the larger societal implications of the knowledge that they gained from the course and were unable to proceed to the issues of engineering macroethics.

Equating social relevance of engineering to the pragmatic and immediate usability of technological innovations was the most common theme in student responses raised in 14 out of the 16 focus groups (87.5%). Elaborating on what they believed to be a good example of the social relevance of engineering, the students listed several topics and experiments in the class, such as drone technology and wind tunnel experiments. One student stated, “…in some remote area in Africa, they’re using drones to actually drop medical supplies because other than that, it takes like a week or something to get a donkey in there so they have drones” (Focus Group 2, 2017). Many students interpreted the social relevance of engineering to the “repurposed” use of military-initiated products and technology in the civilian world. One student summed it up saying:

Most of it (military technology) gets repurposed and used for daily life. There's tons of equipment that was designed for military use and is now every day kind of stuff. They designed Jeeps for military use. Now every other person drives the Jeep around. Duct tape was designed for military use in space. Everyone has duct tape around now. So there's tons of different things that were probably originally in military, but there's more” (Focus Group 1, 2019).

Many responses in this category were notably centered on the practical use, conversion to a commercial product, and immediate availability—though important to engineering—are not necessarily humanitarian or social in nature.

In more than two-thirds of the focus group discussions (11 out of 16), students considered the efficiency and financial benefits of technological innovations (e.g., creating efficient technology to reduce the cost of production) as the prime examples of “social relevance of engineering.” One student claimed, “it’s about how you can make this process more efficient, how can we make this less expensive, how can we release funds from this place to put in this place, is it good in other places…” (Focus Group 1, 2017). Some students were aware of the possibility that engineering innovations not only improve the efficiency of the existing industry but could *lead* a change in society. One student commented, “you can improve some designs with the testing methods learned…You know testing that wind tunnel can improve car performance efficiencies which can affect society” (Focus Group 3, 2018). Another group mentioned that engineering could improve “the overall [energy] infrastructure within cities” based “on renewable energy resources or creating materials that don't erode or corrode to the elements.” They argued for the use of “lighter weight materials to generate wind energy, or even just in terms of the reduction of fossil fuel usage with lighter materials, where we can power cars further on less fuel, or just use lighter cars to utilize different forms of energy” (Focus Group 3, 2018).

Student responses in this category highlighted efficiency and cost reduction using new technologies materials, yet they were largely unable to articulate how such innovations can be reflected upon the larger societal contexts and linked to engineering ethics. Though students were readily able to discuss the technical and pragmatic mission given to the engineering profession, which is both vital and necessary, they struggled to derive the larger ethical concerns surrounding them. They did, however, emphasize the importance of building objects or structures that were safe for use and would do no harm to others, as evidenced by the following quote: “Not to design something that could hurt somebody, so being responsible for what you say it’s supposed to do, so you wouldn’t build a bridge that you knew was unsafe” (Focus Group 2, 2017); “You don’t want to design something and then like somebody misuse it” (Focus Group 3, 2019). Overall, many students’ opinions on the social aspect of their work were centered around efficiency, financial-consciousness, and harm-reductivism, and demonstrated a lack of critical reflection on engineering ethics and the collective role of the profession in the larger society.

### Emerging Macroethics Discourse

Noteworthy is that students in 13 of the 16 focus groups made comments showing their emerging attention to macro-ethics in engineering. While the number of quotes in this category was much less than that of microethics, most focus groups have at least one student addressing this ethical dimension. They explained an eye-opening experience in class when they learned about emerging technologies and discussed their implications. One student expressed her ethical dilemma saying, “tracking and surveillance is probably the biggest thing for me” (Focus Group 3, 2019). The course content—surveillance and tracking technologies—and related open discussions prompted the students to engage in critical reflection as evidenced in one student’s comment: “the class helped me open my eyes to what kind of surveillance systems are out there in the world. I guess the pros and cons of all that kind of stuff” (Focus Group 4, 2019). It is important to note that the students’ raised ethical awareness was often derived from or contextualized in the unique nature of the ENGR 3999 course content that dealt with military technology applications. One student highlighted that point:

That’s in a lot of aspects the most nerve-wracking part of it, is if you’re responsible for designing something that could potentially hurt somebody, be sure that it’s not gonna happen because if it does, what could happen? You just want to be sure that you’ve done everything you can to stop that from happening (Focus Group 2, 2017).

Another student called for a perspective change and re-defined the role of military technology focusing on the prevention of war. He claimed, “a lot of the things the military deals with is kind of humanitarian. So, I guess if you could develop technology that prevents war, that is the ultimate humanitarian effort” (Focus Group 2, 2017). In their end-of-course reflective writing, about a third of the students (21) acknowledged that they had gained “a more evolved ethical mindset about engineering” even though the course did not “explicitly address” engineering ethics. These students provided comments reflecting their growing awareness of the engineering profession’s ethical responsibility to the larger society.

I realize that as engineers, the things we design and manufacture have way greater impact than we can imagine. There inventions/innovations have the potential to change the way people interact with the world or with each other. So, it is important to realize the power a product can have and how it can impact the society (Participant 8 journal, 2019).

I did not know this before, [but] I definitely understand now how much the designs, analyses, and manufacturing of different technologies affect every human on this earth. Although it was not explicitly addressed consistently throughout the semester, I was able to gain a more evolved ethical mindset about engineering. As a result of the ever-evolving technologies, it is imperative that the quality and safety of them be assessed at all times. Each engineer has a moral and ethical obligation to ask for help when needed, and to not approve on a design or technology that may not be properly developed (Participant 11 journal, 2019).

### Apathy to and Disregard of Engineering Ethics

Our analysis showed that students’ awareness of the macro ethical dimension of the course content knowledge varied. Some students acknowledged that they saw “a lot of crossovers” and multiple “indirect” or “broader links” to the larger social implications of each technology covered in the class, yet those opportunities were missed or intentionally ignored. Others could even not recall any conversation that touched the ethical aspects of military technologies. One student recollected, “he (instructor) might've mentioned it like one time, but I can't think of any.” Another student mentioned that there was an implicit expectation to avoid a discussion on engineering ethics: “Engineering morals was more or less the kind of unsaid. Everyone knew what, like you walk in a room, everyone knows, all right, this is what we're doing. Or one of those unsaid things (Focus Group 4, 2019).”

Not surprisingly, there was a reason for the lack of students’ awareness, interest, or willingness to discuss the broader social implications of the course materials. Many students did not consider the social relevance of the course content knowledge an important or even appropriate topic for discussion in the course. They contended that the curriculum should continue to focus on “the technical side” (Focus Group 6, 2017) while avoiding all value-related topics. One student clarified this point saying, “I don't see how that could be [in this class]. I feel like that could be another class, but this class is more just on the technology and instrumentation” (Focus Group 5, 2017). Several other students expressed a similar point claiming the course “wasn’t a social studies course” and “it was really specific technology course” (Focus Group 6, 2017). A dialogue between a graduate student interviewer and four engineering students in Focus Group 4, 2019 illustrates the students’ disinterest and even resistance to discussing ethics in their technical engineering classes. Responding to the interviewer’s question about the relevance of the course content to larger social issues such as social justice or humanitarian causes, students expressed the technical vs. social binary scheme to justify their lack of interest and commitment.

Student 1: This class has been focused on technical knowledge.

Interviewer: Technical knowledge? Okay.

Student 2: Yeah. I can't think of any ethical dilemma. I can't think of any.

Interviewer: Yeah, that’s fair. So, nothing like moral development you learned that shaped your morality as an engineer?

Student 3: No, I mean I don't really go to class to get morals. I go to class to get a technical education.

Interviewer: Yeah, I understand.

Student 3: We didn't spend all this money to come here to learn about right and wrong. What's right and wrong? When you come here to learn more about what we'll be doing for the rest of our lives.

As a result, even though some students were able to address either micro or macro ethical aspects of engineering, others felt that the class was not a place to actively discuss the social relevance of engineering or engineering ethics. In general, though almost all students expressed a provincial knowledge of engineering ethics—especially engineering microethics, many felt that their understanding of the issue remained unchanged and that a further discussion on the topic was unnecessary. In their final reflections, 29 students left a comment that reflected their apathy toward and indifference in discussing engineering ethics. Twenty-seven students explicitly declared they had no perspective change as epitomized by students’ comments: “No, it [the course] has not really changed my views of what an engineer is” (Participant 14 journal, 2017) and “I don’t consider the class to have changed my perception of engineers and the role of engineering in larger society, mainly because it isn’t something that I would think about, even rarely (Participant 14 journal, 2019).” Several students expressed the idea that engineering ethics means only “engineers’ ethics” (Basart & Serra, 2013) and it is personal character, not something that can be facilitated through formal education.

As far as the role an engineer plays in society, I don’t believe this class has changed that. The morals of an engineer are something that isn’t learned necessarily in a classroom. It is who you are as a person and understanding the importance of your role (Participant 2 journal, 2019).

### Searching for Why

Being asked to discuss the social relevance of the course content and possible humanitarian contributions by the engineering profession, some students quickly noticed their own lack of attention to those issues. They started to notice that such lack of attention to engineering ethics was somewhat endemic to the program as a whole and probed a reason why it was so. One student stated:

I feel like as engineers, we don’t really think about that in our classes. We’re always so focused on the engineering aspect of it. You don’t really expand on how is this gonna help society or how does this tie into other areas of the world outside our little world (Focus Group 6, 2017).

Acknowledging the dearth of their exposure to engineering ethics and implications in larger social contexts, one participant mentioned that her peers had been somewhat insulated from the rest of students/programs on campus, which made it difficult for them to “mesh” with those outside of engineering, possibly due to the program’s heavy focus on technical work and the particular style of thinking prevalent in engineering. This student recognized a separation of engineering from other disciplines could have potentially detrimental effects.

Maybe outside of this class somewhere in the program we could have something that does show us how engineers fit into society, because sometimes the thought process on this side of campus is we’re in our own little world, and that’s the other side of campus.

We don’t really feel like it’s inclusive, and I think it goes both ways sometimes, we don’t want to be inclusive. And I think sometimes we don’t feel like we can mesh with people that are outside of our little world because of the way that we think. So I don’t know if that’s something that, somewhere down the line somebody’s going to have to try to integrate a little bit better. But there is a bit of a disconnect there (Focus Group 6, 2017).

Considering that spatial separation between engineering programs and other disciplines, such as humanities and social sciences, is quite common on many college campuses, this student’s comment unveils a critical issue at the structural level of the university, which breeds the sense of separation and mutual exclusion between engineering and non-engineering students. This physical distance makes it hard for students from both sides of campus to experience meaningful interdisciplinary mingling, which is essential to expanding their perspectives.

## Discussion

As technology has emerged as a powerful force in leading our society to an unknown future, we must revisit the social and ethical responsibilities of those who work on the frontline of technological and engineering innovation. This study highlights a critical missing piece in current engineering education—the dearth of exposure and opportunities for students to engage in meaningful discussions about engineering ethics and social responsibilities throughout their course work and extracurricular activities. Findings from our study call for a renewed perspective on engineering ethics that proactively examines the ethical and social consequences of engineers’ work in broader society.

Findings from our study highlight the challenges in facilitating students’ awareness of the two essential dimensions of engineering ethics, macro and microethics. The majority of student responses tended to echo Herkert’s (2001) frame of engineering ethics from the individual perspective, or “microethics”, and lacked the perspective of collective social responsibility from a large-scale institutional standpoint, or “macroethics”. Several authors have emphasized that teaching ethics in engineering education must adequately address both micro and macro ethical domains to give students a working understanding of professional and ethical responsibility, and assure this knowledge leads to long-lasting effects (Bairaktarova & Woodcock, 2016; Herkert, 2001). This result is not surprising. Herkert (2001) reported that although much attention has been given to the teaching of ethical awareness in engineering programs, primarily from a “microethics” framework, much work remains to be done in “macroethics.” Engineering educators largely agree that bridging the gap between “microethics” and “macroethics” is paramount to fulfilling ABET Engineering Criteria 2000 (Criterion 3.f) that emphasizes engineering students a holistic understanding of and commitment to professional and ethical responsibility. This signifies a call for a renewed perspective on teaching ethics in engineering education, one that proactively examines the pedagogies of ethical awareness from both a micro and macro framework.

One of the noteworthy findings in our study was the significant difference in the numbers of students’ responses categorized as “microethics” and “macroethics.” It should be noted that students in the ENGR 3999 class were still able to draw some micro ethical issues concerning the course content and engineering profession even though such discussion was not explicitly prompted by the instructor or TA in the class. This suggests that some engineering course contents may have potential in naturally facilitating students’ ethical awareness and development, especially in microethics. In contrast, such natural facilitation was less obvious in their macroethics development, which, in turn, suggests that macroethics development may require fundamentally different facilitation strategies. Students discovered tension between the “nerve-wracking part” of the course contents and implicit peer pressure not to discuss something beyond the technical aspects of the course contents. Students’ disinterest and refusal to engage in non-technical discussions epitomizes what Cech (2014) contested as the culture of disengagement prevalent in engineering programs. Several previous studies confirmed that facilitating engineering students’ awareness and development in macroethics is the most difficult task (Bucciarelli, 2008, Heckert, 2001), often many well-thought-out intervention programs and specialized engineering ethics courses failed to generate any tangible impact.

It is undeniable that one’s ethical development, especially expounding macroethics, is a very complex and dialogical process; there is no quick solution to facilitate that capacity. Pondering upon multiple facets of an ethical dilemma, engaging in deeper and more critical thinking, and finally expanding one’s ethical horizon inevitably requires constant exposure to several different, challenging points of view. College students immediately and routinely experience tension between their own values and the conflicting values of their peers, as well as of their professors (Matchett, 2008). College students are subsequently engaged in the process of abstract, theoretical thinking, which provides a foundation for “exploring social and ethical challenges from the wider perspectives encountered in higher education” (Brandenberger & Bowman, 2015, p.329). The point—engineers being “in our [their] own little world”—raised by one of the student participants epitomizes one of the most puzzling dilemmas on college campuses, which has been accepted as taken-for-granted and normalized. Engineering students “do not be inclusive… we don’t feel like we can mess with people that are outside of our little world.” In contrast, if we want students to expand the scope of their ethical reasoning, it seems necessary to expose them to different perspectives beyond their disciplines, particularly as research shows that students in social science majors display higher levels of prosocial behavior than their engineering counterparts (Brandenberger & Bowman, 2015). This is not an entirely new idea in higher education or engineering education (Klein, 2010). Many sectors of engineering have long acknowledged the value of interdisciplinary communication and collaboration in generating new ideas and groundbreaking innovations that address the most complex problems in society (Golding, 2012; Klaassen, 2018). Likewise, well-structured interdisciplinary engagement may be a promising avenue in facilitating engineering students’ ethical reasoning (Lee et al., 2020), especially with regards to meaningful development in engineering macroethics.

Despite our careful planning and coordinated effort to draw valid and meaningful interpretations from the given data set, this study presents several important limitations. Four major themes reported in this paper were drawn from a thorough textual analysis that utilized multiple sets of data and researcher triangulation, ensuring their stability and trustworthiness. However, there are limitations to the extent that our findings and conclusions can be transferable to other institutional contexts and their students. First, though focus groups are an effective qualitative data collection method with distinctive advantages, they are not immune to group dynamics, and therefore, participants could have been influenced by their peers and responded in ways to maintain group cohesion. Secondly, ENGR 3999 is a special topics elective course, not required for degree completion by all enrolled engineering students; as such, the students in this study are self-selected limiting the transferability of our findings. The geographical location in the southeast and institutional characteristics (e.g., urban public research institution, engineering program with predominantly White male students) inevitably shaped the students’ experience and perceptions in the ENGR 3999 class. Therefore, findings from this study should be understood with careful consideration of those contextual factors.

## Conclusions

This study contributes to the growing yet limited research exploring engineering students’ perspectives on the non-technical, ethical, and humanitarian aspects of their engineering coursework. Though some students were able to articulate on the societal impact of engineering and engineers’ ethical responsibilities, many others remained within the narrow purview of microethics, and were reluctant or even refused to engage in discussions on macroethics. This work calls attention to the need for engineering programs to develop students with well-rounded competencies, especially a deeper understanding of the interconnectedness of their work with other professions and with society as a whole.
